# H_2_O‐Mg^2+^ Waltz‐Like Shuttle Enables High‐Capacity and Ultralong‐Life Magnesium‐Ion Batteries

**DOI:** 10.1002/advs.202401005

**Published:** 2024-04-06

**Authors:** Xiu‐Fen Ma, Bai‐Qing Zhao, Hongyu Liu, Jing Tan, Hong‐Yi Li, Xie Zhang, Jiang Diao, Jili Yue, Guangsheng Huang, Jingfeng Wang, Fusheng Pan

**Affiliations:** ^1^ National Innovation Center for Industry‐Education Integration of Energy Storage Technology College of Materials Science and Engineering Chongqing University Chongqing 400044 China; ^2^ Materials and Energy Division Beijing Computational Science Research Center Beijing 100193 China; ^3^ National Engineering Research Center for Magnesium Alloys Chongqing University Chongqing 400044 China; ^4^ School of Materials Science and Engineering Northwestern Polytechnical University Xi'an 710072 China; ^5^ National Key Laboratory of Advanced Casting Technologies Chongqing University Chongqing 400044 China

**Keywords:** cathode material, H_2_O molecules, hydrated vanadium oxide, magnesium‐ion battery, migration kinetics

## Abstract

Mg‐ion batteries (MIBs) are promising next‐generation secondary batteries, but suffer from sluggish Mg^2+^ migration kinetics and structural collapse of the cathode materials. Here, an H_2_O‐Mg^2+^ waltz‐like shuttle mechanism in the lamellar cathode, which is realized by the coordination, adaptive rotation and flipping, and co‐migration of lattice H_2_O molecules with inserted Mg^2+^, leading to the fast Mg^2+^ migration kinetics, is reported; after Mg^2+^ extraction, the lattice H_2_O molecules rearrange to stabilize the lamellar structure, eliminating structural collapse of the cathode. Consequently, the demo cathode of Mg_0.75_V_10_O_24_·nH_2_O (MVOH) exhibits a high capacity of 350 mAh g^−1^ at a current density of 50 mA g^−1^ and maintains a capacity of 70 mAh g^−1^ at 4 A g^−1^. The full aqueous MIB based on MVOH delivers an ultralong lifespan of 5000 cycles The reported waltz‐like shuttle mechanism of lattice H_2_O provides a novel strategy to develop high‐performance cathodes for MIBs as well as other multivalent‐ion batteries.

## Graphical abstract

Universal Mg^2+^‐H_2_O waltz‐like shuttle mechanism: the lattice H_2_O molecules in the lamellar structure of Mg_0.75_V_10_O_24_·nH_2_O (MVOH) coordinate and co‐migrate with Mg^2+^ during discharging, meanwhile, they adaptively rotate and even flip to assist the migration of Mg^2+^, which facilitates the Mg^2+^ migration kinetics. After Mg^2+^ extraction, the lattice H_2_O molecules rearrange as supporting pillars to stabilize the lamellar structure of MVOH.

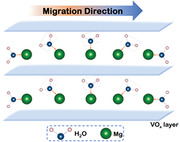

## 1 Introduction

Li‐ion batteries (LIBs) have revolutionized human life and modern industries, whose further development is limited by the Li resource shortage and the low safety caused by sharp Li dendrites.^[^ ^1^, ^2^, ^3^ ^]^ Despite the advantages of magnesium‐lithium hybrid batteries (MLHBs) in terms of energy density, cycle life, charging and discharging speeds, safety, etc., they still have the dendrite problem of lithium batteries.^[^ ^4^, ^5^ ^]^ Mg‐ion batteries (MIBs) are promising next‐generation energy storage electrochemical devices due to the abundance of Mg resources and the high safety endowed by the inhibition of sharp dendrites.^[^ ^6^, ^7^ ^]^ Similar to charge shuttling by Li^+^ ions in LIBs, the energy storage of MIBs relies on the charge shuttling of Mg^2+^ ions. Due to the high charge density of Mg^2+^ ions, it is usually believed that the Mg^2+^‐cathode interaction is purely ionic and very strong, and Mg^2+^ ions must individually diffuse long distances through the cathode materials, leading to the sluggish kinetics of Mg^2+^ migration and collapse of cathode crystal structure.^[^ ^8^, ^9^ ^]^ Hence, MIBs suffer severely from the low capacity and the short cycle life. To solve this challenge, recent excellent work has used carbon nanotube frameworks as electronic transport channels to enhance the Mg^2+^ diffusion kinetics, thereby improving the capacity of MIBs, but the long‐term cycling performance is still not satisfactory.^[^ ^10^ ^]^

The Mg^2+^ ions, unlike other metal ions including Li^+^, have strong interactions with H_2_O molecules and the resulting Mg─O bonds can be as short as 2.04 Å, which makes the migration of Mg^2+^ distinguished with the presence of lattice H_2_O molecules. The lattice H_2_O molecules were reported to rapidly shuttle H^+^ in their immobilized network via the strong interaction between H^+^ and H_2_O molecules, which suggests a possible similar ability to shuttle Mg^2+^.^[^ ^11^ ^]^ Recent studies disclosed that the lattice H_2_O molecules in the cathode can 1) partially shield the electrostatic interaction between Mg^2+^ and cathode; and 2) stabilize the lamellar structure as pillars, either in the aqueous or organic MIBs.^[^ ^12^, ^13^, ^14^ ^]^ The solvent H_2_O molecules can even be inserted into the narrow spacing of lamellar cathode prior to cations, which impel the following co‐(de)insertion of Mg^2+^/H^+^ in aqueous MIBs.^[^ ^15^ ^]^ However, it remains unanswered whether H_2_O molecules co‐migrate with Mg^2+^ in the cathode and how to use the Mg^2+^‐H_2_O interaction to increase the capacity and cycle life of MIB cathodes.

In order to provide enough lattice H_2_O molecules in cathode, we design the lamellar Mg_0.75_V_10_O_24_·4H_2_O (denoted as MVOH) cathode with a large spacing of 13.9 Å, which contains abundant lattice H_2_O to fast shuttle Mg^2+^ for high capacity and pillar the lamellar structure for long cycle life; meanwhile, the mixed valence of V^4+^ and V^5+^ can facilitate the 3d electron hopping and thus increase the electronic conductivity of MVOH, further ensuring the high capacity. Our ab initio calculations and molecular dynamics simulations suggest an H_2_O‐Mg^2+^ waltz‐like shuttle mechanism in our MVOH cathode. Unlike immobilization of H_2_O molecules during shuttling H^+^, the lattice H_2_O molecules coordinate and co‐migrate with Mg^2+^ during discharging, which realizes the waltz‐like shuttle mechanism via adaptively rotating or even flipping to accelerate Mg^2+^ migration kinetics; after Mg^2+^ extraction during charging, the lattice H_2_O molecules rearrange to pillar the lamellar structure for stability improvement. Results show that the H_2_O‐Mg^2+^ waltz‐like shuttle mechanism excludes the co‐(de)insertion of H^+^ with Mg^2+^, which eliminates electrolyte decomposition, further increasing the cycling life of MIBs. Consequently, the MVOH cathode exhibits a remarkably high discharge capacity of 350 mAh g^−1^ at a current density of 0.05 A g^−1^ and high‐rate capability (70 mAh g^−1^ at 4 A g^−1^); the aqueous MIB with the MVOH cathode delivers an ultralong cycling life of 5000 cycles and a high energy density of 67 Wh kg^−1^.

## 2 Results and Discussion

### 2.1 Characterization of 3D MVOH Cathode

**Figure 1:**
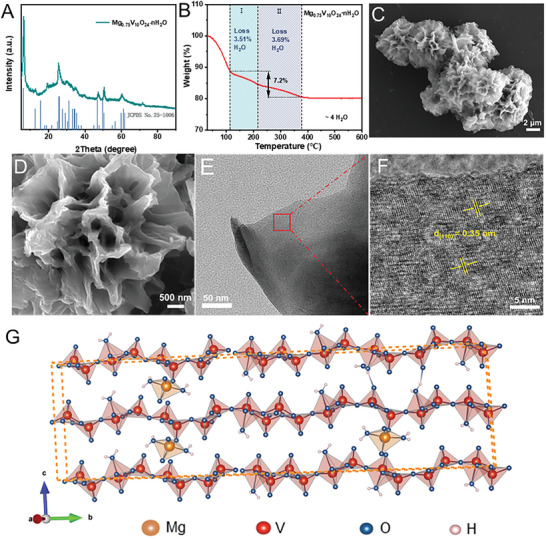
Physicochemical characterization of the MVOH cathode: A) XRD pattern. B) TGA profile. C,D) SEM images. E,F) TEM images. G) Crystal structure of MVOH.

Mg_0.75_V_10_O_24_·4H_2_O (denoted as MVOH) was obtained via a simple one‐step precipitation method. In the X‐ray powder diffraction (XRD) pattern (**Figure** **1**A), the sharp diffraction peak located at 2*θ* = 6.2° relates to the (002) planes, whose corresponding interlayer spacing is as large as 13.9 Å according to the Bragg equation, much larger than that of the reported hydrated Mg_x_V_5_O_12_ (11.9 Å).^[^ ^14^ ^]^ The large interlayer spacing provides potentially abundant lattice H_2_O molecules. The stoichiometric formula of MVOH is determined to be Mg_0.75_V_10_O_24_·4H_2_O by combining the inductively coupled plasma‐optical emission spectroscopy (ICP‐OES, Table S1, Supporting Information) and thermogravimetric analysis (TGA, Figure 1B). Specifically, the 7.2% weight loss is attributed to the loss of lattice H_2_O, according to which there are 4 lattice H_2_O molecules for each unit cell of Mg_0.75_V_10_O_24_. In the X‐ray Photoelectron Spectroscopy (XPS) survey (Figure S1, Supporting Information), the two peaks at 517.4 and 524.9 eV correspond to the V2p_3/2_ and V2p_1/2_ levels of V^5+^, respectively, while the peaks at 516.1 and 523 eV relate to the V2p_3/2_ and V2p_1/2_ levels of V^4+^.^[^ ^16^ ^]^ According to the peak deconvolution, 65% of V exists in the form of V^5+^ while 35% of V exists in the form of V^4+^; the average valence of V is thus calculated to be +4.64, which is consistent with the chemical formula of Mg_0.75_V_10_O_24_·4H_2_O. The existence of V^4+^ indicates that Mg^2+^ has been pre‐inserted into the interlayer of vanadium oxide and the valence of V has thus been partially reduced (Figure S1C, Supporting Information).^[^ ^17^ ^]^ The co‐existence of V^5+^ and V^4+^ in MVOH provides opportunities for the 3*d* electrons to hop from occupied 3*d* orbitals of V^4+^ to empty 3*d* orbitals of V^5+^, which increases the electronic conductivity of MVOH and thus its capacity.^[^ ^18^, ^19^, ^20^ ^]^ Furthermore, three distinct components can be observed in the O 1*s* core level, which are attributed to the O atoms in V─O (530.2 eV), Mg‐O (531.4 eV), and H_2_O (533.2 eV), respectively (Figure S1D, Supporting Information).^[^ ^21^ ^]^ The Raman spectrum and Fourier‐transformed infrared (FT‐IR) spectra of MVOH further verify its lamellar structure constituted by V─O skeleton layers (Figures S4 and S5, Supporting Information).

Scanning electron microscopy (SEM) images of MVOH display a nanoflower morphology, along with homogeneity and high yield, and the petal thickness is ≈60–70 nm (Figure 1C,D). The nanoflower morphology greatly increases the specific surface area of the MVOH and shortens the diffusion path of Mg^2+^ from bulk solution to active sites, which thus accelerates the insertion/extraction of Mg^2+^. Transmission electron microscopy (TEM) images further show the nanoflower structure of MVOH (Figure S2, Supporting Information). In the high‐resolution TEM (HRTEM) images (Figure 1E,F), a lattice spacing of 0.35 nm is observed, which corresponds to the (110) lattice planes of MVOH. According to scanning transmission electron microscopy‐Energy‐dispersive X‐ray spectroscopy (STEM‐EDS) mapping (Figure S3, Supporting Information), the Mg, V and O elements distribute uniformly in MVOH nanoflowers, and the atomic ratio of Mg to V is ≈1.0:13.2 (Table S2, Supporting Information), which is consistent with the ICP‐OES results, further confirming the composition of Mg_0.75_V_10_O_24_·4H_2_O. The N_2_ adsorption/desorption isotherm of MVOH exhibits a type‐IV isotherm and a distinct H3 hysteresis loop within the relative pressure range of 0.80–0.98, which suggests that the pores in the nanoflowers consist of narrow slits or crack‐shaped holes formed by the accumulation of flake particles (Figure S6A, Supporting Information).^[^ ^22^ ^]^ According to the fitted adsorption/desorption curve in the relative pressure range of 0–0.26 (inset in Figure S6A, Supporting Information), the specific surface area of MVOH nanoflowers is 33.07 m^2^ g^−1^, which is higher than those of other vanadium oxides.^[^ ^12^, ^23^ ^]^ The MVOH nanoflowers exhibit an average pore diameter of 19.23 nm with a total pore volume of 0.12 cm^3^ g^−1^. The pore size distribution of the MVOH nanoflowers (Figure S6B, Supporting Information) centralizes on the macropores ≈52 nm, suggesting the hierarchical pore structure of MVOH nanoflowers, which can enhance the permeability of Mg^2+^ between the MVOH cathode and the electrolyte, and thus increase the capacity of MVOH cathode.

To further obtain the MVOH crystalline structure with lattice H_2_O molecules mediating Mg^2+^ conduction, density functional theory (DFT) calculations are performed on the basis of structural characterization results including XRD, TGA, and XPS. MVOH has a lamellar structure constituted by VO_x_ polyhedra layers that consist of VO_5_ rectangular pyramids and VO_6_ octahedra (Figure 1G; Figure S7, Supporting Information). The lattice H_2_O molecules distribute in the interlayer space and combine with the VO_x_ polyhedra layers either indirectly or directly. The indirectly combined (IDC) lattice H_2_O molecules combine with VO_x_ layers via pre‐intercalated Mg^2+^ cations; two such IDC lattice H_2_O molecules coordinate with one Mg^2+^ cation, which simultaneously coordinates with O atoms in VO_5_ rectangular pyramids, leading to the formation of MgO_5_ hexahedra between VO_x_ layers. The resulting MgO_5_ hexahedra also support the lamellar structure of MVOH as pillars, further stabilizing the MVOH crystal structure. The directly combined (DC) lattice H_2_O molecules involve coordination with 1) V^4+^/V^5+^ cations, leading to the formation of VO_6_ octahedra, denoted as DC‐1; and 2) O atoms in VO_5_ rectangular pyramids via hydrogen bonds (DC‐2). These DC lattice H_2_O molecules combine directly with either the downside surface of upper VO_x_ layers or the upside surface of lower VO_x_ layers in the interlayer space, which connect the adjacent two VO_x_ layers by hydrogen bonds and function as supporting pillars to stabilize the lamellar structure of MVOH, and as targets to facilitate connectivity with Mg^2+^. These DC lattice H_2_O molecules are flexible and abundant to transfer newly inserted Mg^2+^ via the waltz‐like shuttle mechanism.

### 2.2 Mg^2+^ Conduction for Fast Electrochemical Kinetics

**Figure 2:**
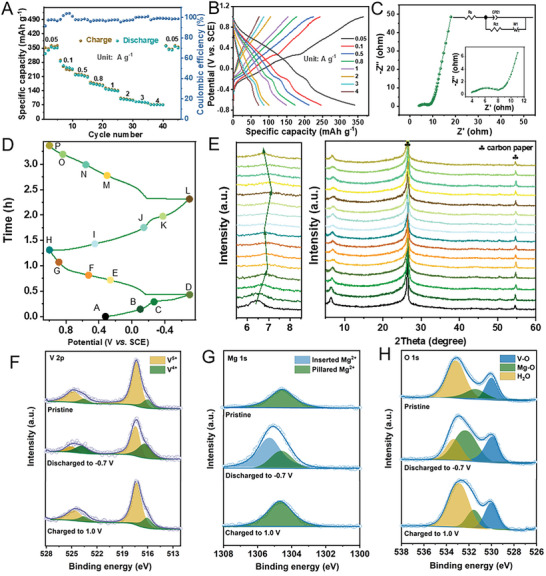
Electrochemical performance and electrochemical reaction mechanism of the MVOH cathode: A) Rate capability plots. B) Discharge‐charge curves of MVOH at different rates. C) EIS spectrum. D,E) Ex situ XRD patterns at different discharge/charge states of the MVOH cathode. F) Ex situ high‐resolution XPS spectra of the MVOH cathode at pristine, fully discharged, and fully charged states with V 2*p* core level. G) Ex situ high‐resolution XPS spectra of the MVOH cathode at pristine, fully discharged, and fully charged states with Mg 1*s* core level.

In order to evaluate the intrinsic Mg^2+^ storage capability of the MVOH nanoflowers as MIB cathode, a three‐electrode configuration was assembled in ambient air with a platinum electrode as the counter electrode and a saturated calomel electrode (SCE) as the reference electrode in 2 m Mg(CF_3_SO_3_)_2_ aqueous solution. The initial three cycles of cyclic voltammetry (CV) curves within a potential window of −0.7–1.0 V versus SCE at a scanning rate of 1 mV s^−1^ (Figure S8, Supporting Information). In the cathodic scan, a broad peak at 0.12 V and a sharp reduction peak at −0.20 V are observed, which correspond to the reduction of V and the insertion of Mg^2+^; in the anodic scan, three oxidation peaks are observed at −0.11, 0.08, and 0.30 V, which correspond to the oxidation of V^4+^ during the stepwise extraction process of Mg^2+^.^[^ ^24^, ^25^, ^26^, ^27^ ^]^ The CV curves of the initial three cycles differ slightly, suggesting excellent cyclic reversibility of the MVOH nanoflowers. At a current density of 0.05 A g^−1^, the MVOH cathode exhibits a remarkably high discharge capacity of 350 mAh g^−1^ (**Figure** **2**A), which surpasses those of reported Li_3_V_2_(PO_4_)_3_ (115.1 mAh g^−1^), MgMn_2_O_4_ (166 mAh g^−1^), and δ‐MnO_2_ (220 mAh g^−1^) as MIB cathodes (Table S3, Supporting Information). This remarkably high capacity implies fast migration kinetics of Mg^2+^ in MVOH nanoflowers with abundant lattice H_2_O. When the current density increases to 1 A g^−1^ (Figure 2A,B), a capacity of 149 mAh g^−1^ is obtained, which is about two times higher than that of the reported Mg_x_V_5_O_12_ cathode.^[^ ^14^ ^]^ With the current density increasing to 4 A g^−1^, a desirable capacity of 70 mAh g^−1^ can be consistently achieved, suggesting a high‐rate capability of the MVOH cathode. Upon recovering the current density to 0.05 A g^−1^, the specific capacity of MVOH nanoflowers promptly recovers to 350 mAh g^−1^, indicating a longer cycling stability of the MVOH cathode. The galvanostatic charge/discharge curves at different current densities present similar charge and discharge voltage plateaus at 0.3 and −0.2 V, respectively (Figure 2B), highlighting the fast Mg^2+^ migration kinetics and good rate capability of MVOH nanoflowers cathode. Even after undergoing 100 cycles at a high current density of 3 A g^−1^, the discharge capacity still maintains 64% of its initial value (Figure S9, Supporting Information), whose retention rate is high for beaker cells, since slight dissolution of vanadate is commonly observed in the presence of abundant aqueous solution.^[^ ^28^ ^]^ The high capacity and cycling stability of the MVOH cathode is achieved by the pillaring function of abundant lattice H_2_O molecules.

To quantitatively investigate the Mg^2+^ conduction kinetics in the MVOH cathode, CV tests are performed at different scan rates between 0.2 and 1.0 mV s^−1^ (Figure S10, Supporting Information). The CV curves exhibit similar shapes and redox peaks, indicating fast migration kinetics and highly reversible insertion/extraction of Mg^2+^. The Mg^2+^ storage kinetics is evaluated by utilizing the quantitative equation *i* = aν^b^, which establishes a relation between the peak current (*i*) and the scan rate (*v*). Results show that the Mg^2+^ storage process is synergistically controlled by both Faradaic intercalation and pseudocapacitance mechanisms (Figure S10A,B, Supporting Information).^[^ ^29^ ^]^ The pseudocapacitive behavior contributes to ≈62.50–74.31% of the total capacity at a scan rate of 0.2–1.0 mV s^−1^ (Figure S10C,D, Supporting Information). These results indicate that the high capacity of the MVOH cathode primarily originates from the pseudocapacitive redox reactions on the MVOH surface, which effectively accounts for the exceptional reversibility and rate capability of the MVOH nanoflowers. Electrochemical impedance spectroscopy (EIS) tests are carried out to further investigate the Mg^2+^ conduction kinetics in the MVOH cathode (Figure S11, Supporting Information). The diffusion coefficient of Mg^2+^ (*D*_Mg_) in the MVOH cathode is calculated to be 1.08 × 10^−9^ cm^2^ s^−1^, which is 1–3 order(s) of magnitude greater than those of conventional cathodes (Table S4, Supporting Information). This high *D*_Mg_ indicates fast migration kinetics inside the MVOH crystal. The charge transfer resistance (*R*_ct_) at the cathode/electrolyte interface is fitted to be a remarkably low value of 5.29 Ω, indicating fast crossing of Mg^2+^ at the solid/liquid interface. The fast bulk migration and interface crossing facilitate the surface pseudocapacitive reactions, leading to the remarkably high capacity and good rate capability of the MVOH cathode.

To investigate the Mg^2+^ conduction mechanism of the MVOH cathode, systematic ex situ characterizations are performed at various charged/discharged states in the initial cycle (Figure 2D). In the ex situ XRD patterns (Figure 2E), new diffraction peaks do not appear throughout the continuous Mg^2+^ insertion/extraction. Meanwhile, the peak intensities change little. These results suggest the absence of phase transformations during the insertion/extraction of Mg^2+^ in the MVOH cathode. During the initial discharging (Points A‐D), the diffraction peak of the (002) lattice plane shifts from 6.5° to 6.9° with Mg^2+^ inserted into the MVOH nanoflowers, signifying shrinkage of the interlayer spacing from 13.6 to 12.8 Å. The decrease in interlayer spacing is attributed to the strong electrostatic interaction between the inserted Mg^2+^ ions and the VO_x_ polyhedra layers.^[^ ^30^, ^31^ ^]^ Due to the “pillar effect”^[^ ^32^ ^]^ of both lattice H_2_O molecules and pre‐intercalated Mg^2+^ ions in the interlayer, the crystal structure of MVOH can be well preserved and the interlayer spacing shrinks by only 7.5%. During the charging process (Points D‐H), the (002) peak gradually shifts toward the left to 6.8°, which corresponds to an interlayer spacing of 13.0 Å. This result indicates that part of the inserted Mg^2+^ cations are retained in the interlayer between VO_x_ layers. During the second discharging process (Points H‐L), the (002) peak shifts toward the right to 7.0°, and shifts back to 6.8° in the second charging process (Points L‐P). After the second discharging/charging cycle, the interlayer spacing recovers to 13.0 Å. This indicates that a portion of Mg^2+^ is permanently inserted into the interlayer of MVOH after the first discharge, further strengthening the structural stability of the V_10_O_24_ skeleton. Ex situ TEM characterizations show that the lattice spacing of the (110) planes in the fully discharged MVOH cathode decreases from 0.35 nm (pristine) to 0.20 nm (Figure S12A,B, Supporting Information). When fully charged, the lattice spacing of the (110) crystal planes increases to 0.33 nm, indicating a limited variation of crystal structure (Figure S12C, Supporting Information).

Ex situ XPS spectra of V 2*p*, Mg 1*s*, and O 1*s* were acquired from the pristine, fully discharged, and fully charged MVOH cathodes, respectively. In Figure 2F, the V 2*p* core‐level spectrum of the pristine MVOH cathode exhibits a small amount of V^4+^, which is attributed to the incorporation of pre‐intercalated Mg^2+^ cations in MVOH. Notably, the component of V^4+^ increases significantly after full discharge, while the proportion of V^5+^ decreases. These results indicate that the reduction of V^5+^ takes place during the insertion of Mg^2+^ into the MVOH cathode, providing a high discharge capacity. When the MVOH cathode gets fully charged, the valence change of V is reversed and V^4+^ has been oxidized to V^5+^ to maintain a content similar to pristine MVOH. For the Mg 1*s* core level (Figure 2G), the pristine MVOH cathode shows a signal peak at 1304.7 eV, which is attributed to the pre‐intercalated Mg^2+^ ions that function as pillars in the interlayer. The fully discharged MVOH cathode exhibits a new signal peak at a higher binding energy of 1305.3 eV, together with the signal at 1304.7 eV, which is related to the insertion of the Mg^2+^ cations into the interlayer during discharging. In comparison to the pre‐intercalated Mg^2+^, the interaction between the newly inserted Mg^2+^ and the VO_x_ polyhedra layers is weaker.^[^ ^33^ ^]^ The fully charged MVOH cathode exhibits a slightly higher signal at 1304.7 eV than the pristine one, indicating the slight residual of newly inserted Mg^2+^ in the interlayer of MVOH and the pillar function of residual Mg^2+^ cations for structural stabilization. Figure 2H shows the O 1*s* core levels of MVOH, in which three distinct O atoms are observed, including the O atoms in the VO_x_ polyhedra layers (V─O, 530.4 eV), the O atoms coordinated with Mg^2+^ (Mg─O─V and Mg─OH_2_, 531.7 eV), and the O atoms in DC lattice H2O in the interlayer (H_2_O, 533.1 eV).^[^ ^21^ ^]^ When fully discharged, the Mg─O signal increases noticeably while the signal of DC lattice H_2_O decreases significantly. This observation indicates coordination between the DC lattice H_2_O and the newly inserted Mg^2+^ cations.^[^ ^34^ ^]^ Particularly, the binding energy of the DC lattice H_2_O is increased in the discharged MVOH cathode in comparison to the pristine cathode. This indicates that the remaining DC lattice H_2_O molecules in the interlayers are closer to a “free” physisorbed state, rather than being chemically bonded with the skeleton, which implies that these H_2_O molecules shuttle Mg^2+^ during discharging.^[^ ^35^ ^]^ At the fully charged state, all O signals in the MVOH cathode return to their initial states as in the pristine cathode, indicating a reversible intercalation mechanism involving the rearrangement of DC lattice H_2_O molecules in the interlayer of MVOH.

### 2.3 Mg^2+^ Transfer via the Waltz‐Like Shuttle Mechanism in MVOH

In order to investigate the microscopic migration mechanism of Mg^2+^ in MVOH, ab initio calculations based on DFT are conducted (see computational details in the Experimental Section of the Supporting Information). Based on the determined pre‐intercalated structure of MVOH, we analyzed and identified the energetically favorable sites for Mg^2+^ to occupy. Further, we locate the nearest neighboring site for Mg^2+^ to hop to based on symmetry analysis and rigorous structural relaxations. According to the relaxed structures, we discover the waltz‐like Mg^2+^ shuttle mechanism by DC lattice H_2_O molecules in MVOH.

**Figure 3:**
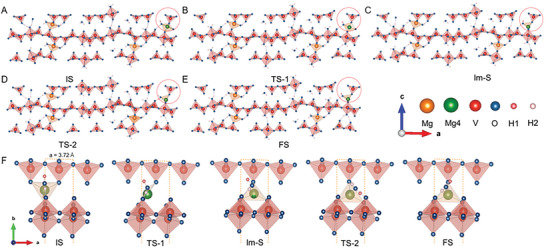
The structural change of MVOH in the *a* direction projection during the H_2_O‐Mg^2+^ waltz‐like shuttle: A) Initial state. B) Transition state of 1. C) Intermediate state. D) Transition state of 2. E) Final state. F) Migration of Mg^2+^ along the *a* direction in the interlayer of MVOH.

**Figure** **3**A–E shows the structural change of MVOH (projected along the *a* direction) during the H_2_O‐Mg^2+^ waltz‐like shuttle. Figure 3F summarizes the waltz‐like Mg^2+^ shuttle process of DC lattice H_2_O molecules along the *a* direction in the interlayer of MVOH. In the initial state (IS) of MVOH with Mg^2+^ inserted, the Mg^2+^ cation is located at the left edge of the lattice unit (dashed rectangle), which coordinates with five O atoms to form MgO_5_ hexahedron, including one O atom from a DC‐2 lattice H_2_O molecule; such coordination converts this DC‐2 lattice H_2_O molecule into an IDC lattice H_2_O molecule. As Mg^2+^ migrates along the *a* direction, a MgO_4_ square forms with Mg^2+^ in the square center (TS‐1). As Mg^2+^ migrates further to the mirror plane of the lattice unit (Im‐S), Mg^2+^ coordinates with the O atom at the right edge of the lattice unit, leading to the formation of MgO_4_ tetrahedron. Meanwhile, this IDC H_2_O molecule coordinated with Mg^2+^ migrates together with Mg^2+^, as shown from IS to Im‐S in Figure 3F. Interestingly, the coordinated IDC lattice H_2_O molecule flips with its red H atom (H1) moving upside down to generate a H‐bond with one O atom at the right edge of the lattice unit (TS‐2); Mg^2+^ moves inside vertically away from the screen along the *b* direction. Thereafter, this coordinated IDC H_2_O molecule flips again with the pink H atom (H2) moving upside down to generate an H‐bond with the O atom at the left edge of the lattice unit, whereas the red H1 atom distributes in the right part of the lattice unit (FS); Mg^2+^ moves back closer to the screen but proceeds along the *a* direction. After flipping this coordinated IDC H_2_O molecule twice, Mg^2+^ adjusts its location along the *b* direction but proceeds further along the *a* direction. The cooperative rotation and flipping of the new IDC H_2_O molecules coordinated with inserted Mg^2+^ cations facilitate the Mg^2+^ migration in MVOH, which demonstrates the waltz‐like Mg^2+^ shuttle mechanism of lattice H_2_O molecules in the MVOH cathode. As mentioned above, the ex situ XPS characterizations (Figure 2H) uncovered a rearrangement of structural H_2_O molecules in the interlayer during charging. Combining these facts, it is deduced that after extraction of Mg^2+^ from MVOH, the Mg^2+^─H_2_O bonds break, and the released H_2_O molecules distribute in a configuration with high energy. In order to minimize the system energy, these released H_2_O molecules rearrange to generate H‐bonds with VO_x_ polyhedra layers, which stabilize the lamellar structure of MVOH as their initial distribution configuration.

**Figure 4:**
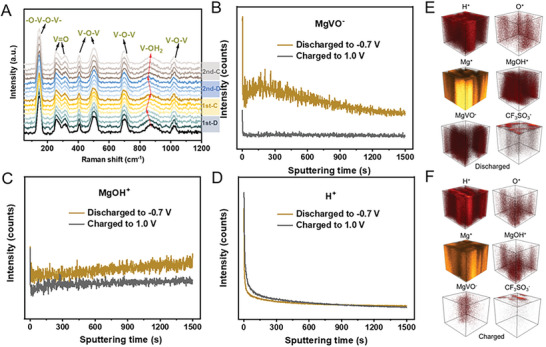
Electrochemical reaction mechanism of the MVOH cathode: A) In situ Raman spectrum at different discharge/charge states of the MVOH cathode. B–D) Depth profiles. E,F) TOF‐SIMS images of H^+^, O^+^, Mg^+^, MgOH^+^, MgVO^−^, and CF_3_SO_3_^−^ of fully discahged and charged electrode.

To verify the Mg^2+^ shuttle function of lattice H_2_O molecules, in situ Raman characterization of MVOH cathode during discharging/charging has been conducted (**Figure** **4**A). The Raman shift of scattering peak at 871 cm^−1^, corresponding to the V─OH_2_ bonds, variates at different discharged/charged states. In the discharging process, the Raman shift decreases from 871 to 850 cm^−1^, which suggests an increase in the electron cloud density of the V─OH_2_ bonds. This highly agrees with the Mg^2+^ shuttle function of lattice H_2_O molecules. The DC‐2 lattice H_2_O molecules coordinate with Mg^2+^ cations to shuttle, generating the MgO_5_ rectangular pyramids in the interlayer, while the DC‐1 lattice H_2_O molecules maintain in the VO_6_ octahedra via the V─OH_2_ bonds to stabilize the lamellar structure of MVOH (Figure S13, Supporting Information). However, the coordination of Mg^2+^ with O atoms from the VO_6_ octahedra causes a more positive charge of V to contribute to the V─OH_2_ bonds, leading to the shorter V─OH_2_ bond length and thus the higher electron cloud density. According to the ab initio calculation results, the pristine MVOH has a V─OH_2_ bond length of 2.58 Å; this bond length in IS‐state MVOH decreases to 2.29 Å and decreases further to 2.26 Å in the FS‐state MVOH, as labeled in Figure S13 (Supporting Information). In the charging process, the Raman shift of V─OH_2_ bonds returns to 871 cm^−1^, which implies the bond length recovery of V‐OH_2_ bonds, indicating the arrangement recovery of DC lattice H_2_O molecules in MVOH after Mg^2+^ extraction. This is in good accordance with ex situ XPS characterization results in Figure 2H. To further verify the Mg^2+^ shuttle function of lattice H_2_O molecules, the time of flight‐secondary ion mass spectrometry (TOF‐SIMS) is employed to analyze the fully discharged and charged MVOH cathode (Figure 4B–F; Figure S14, Supporting Information). The much higher intensity of MgVO^−^ signal in discharged MVOH than that in charged MVOH suggests successful insertion of Mg^2+^ during discharging (Figure 4B,E,F). The MgOH^+^ signal in discharged MVOH is significantly higher than that in charged MVOH, which demonstrates the strong coordination of lattice H_2_O molecules with Mg^2+^ inserted during discharging, verifying the H_2_O─Mg^2+^ shuttle mechanism (Figure 4C,E,F). Furthermore, the H^+^ signal intensities and distribution in discharged and charged MVOH are almost the same, which indicates that the H_2_O‐Mg^2+^ waltz‐like shuttle mechanism excludes the co‐insertion or co‐extraction of H^+^ together with Mg^2+^ (Figure 4D–F).

### 2.4 Mg^2+^ Transfer Path and Energy Barrier

**Figure 5:**
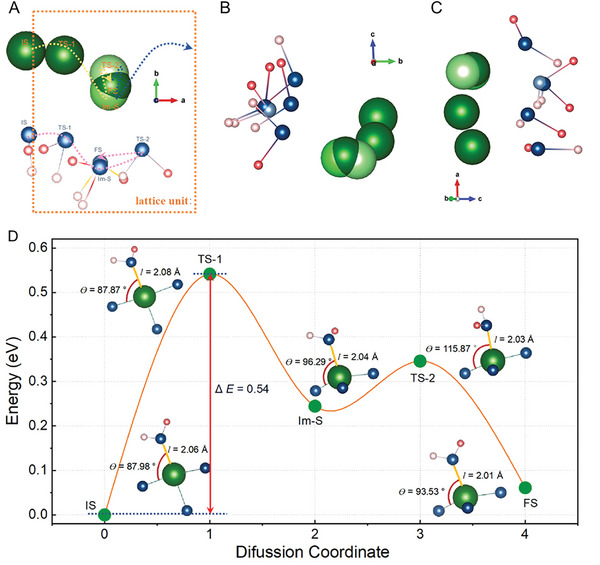
Migration of Mg^2+^ along the *a* direction in the interlayer of MVOH: A–C) The zig‐zag migration path of Mg^2+^ in a lattice unit (dashed rectangle) in the different planes of MVOH. D) Minimum energy path of Mg^2+^ ion migration in the MVOH crystal.

Nudged elastic band (NEB) simulations^[^ ^36^ ^]^ are performed to derive the minimum energy path for the atomic hop of Mg^2+^ between its favorable site and the nearest neighboring site. **Figure** **5**A–C shows the zig‐zag migration path of Mg^2+^ in a lattice unit (dashed rectangle) in the *ab* plane of MVOH, which benefits from the large interlayer along the *c* direction of MVOH. As Mg^2+^ migrates in the left part of the lattice unit (from IS to FS), its coordinated H_2_O molecule migrates together, which simultaneously adjusts the orientation of itself via rotation or even flipping to cooperatively assist the zig‐zag migration of Mg^2+^, leading to the stabilization of each step of Mg^2+^ migration (shown in Movie S1, Supporting Information). Similarly, Mg^2+^ can migrate axisymmetrically in the right part of the lattice unit via evolution among the states of FS→TS‐2→Im‐S→TS‐1→IS; by this way, the Mg^2+^ cation completes the migration in one lattice unit of MVOH. These results disclose the critical role of the coordinated lattice H_2_O molecules in shuttling Mg^2+^ cation to migrate in the interlayer of MVOH.

Figure 5D shows that the double flipping of the H_2_O molecule minimizes the system energy and leads to closer coordination with Mg^2+^ since the length of the Mg─OH_2_ coordination bond decreases from 2.04 Å (Im‐S state) to 2.01 Å (FS state). From the IS to the FS state, the H_2_O molecule coordinates with Mg^2+^ more closely and more strongly via its adaptive rotation and flipping, which makes the Mg^2+^ cation ready for the axisymmetrical round of H_2_O shuttled zig‐zag migration in the right half part of the lattice unit (dashed blue track in Figure 5A). In addition, the energy barrier of the H_2_O shuttled zig‐zag migration is as low as 0.54 eV, which indicates the fast migration kinetics of Mg^2+^ in MVOH (Table S5, Supporting Information). This result agrees very well with the determined high capacity and good rate capability of the MVOH cathode. Furthermore, the volume change of MVOH before and after Mg^2+^ insertion is investigated (Figure S15, Supporting Information). After Mg^2+^ insertion, the lattice constant *a* expands by 0.27% while the lattice constant *b* expands by 0.35%, with the lattice constant *c* shrinking by 0.63%. The total volume of one lattice unit shrinks as low as 0.012%, which is negligibly small. These data demonstrate the high structure stability of MVOH during charging/discharging, which well explains the long cycling life of MVOH cathode. Therefore, due to the waltz‐like shuttle of coordinated lattice H_2_O molecules, Mg^2+^ can migrate in the interlayer of MVOH with fast kinetics and minor lattice change, which endows MVOH to exhibit high capacity, good rate capability, and long cycling life for Mg^2+^ storage.

### 2.5 Electrochemical Performance of MIBs with MVOH Cathode

**Figure 6:**
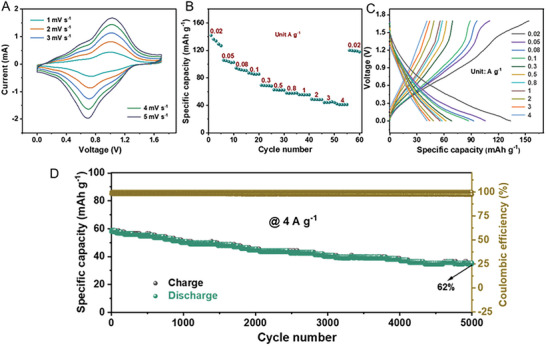
Electrochemical performance of PTCDA//MVOH full cell: A) CV curves at different scan rates. B,C) Rate capability and GCD curves at different current densities. D) Cycling stability at 4 A g^−1^.

To further explore the excellent performance of the MVOH cathode, we fabricate a full MIB with the MVOH nanoflowers as the cathode, perylene‐3,4,9,10‐tetracarboxylic dianhydride (PTCDA) as anode, and 2 m Mg(CF_3_SO_3_)_2_ aqueous solution in PEG/H_2_O volume ratio of 1:1 as electrolyte (Figure S16, Supporting Information). The CV curves in **Figure** **6**A depict the performance of the full cell within the potential range of 0–1.7 V at scan rates of 1–5 mV s^−1^. A pair of cathodic/anodic peaks are located at 0.73 and 1.01 V, which correspond to the insertion and extraction of Mg^2+^ cations in the MVOH cathode, respectively. In addition, the shape of the CV curves changes little as the scan rate increases, indicating highly reversible Mg insertion/extraction and good rate capability of the full cell. Figure 6B,C shows the rate performance of the PTCDA//MVOH full cell at various current densities, along with the corresponding galvanostatic charge‐discharge (GCD) curves. The full cell delivers a high discharge capacity of 133 mAh g^−1^ at a current density of 0.02 A g^−1^. Remarkably, even with a 200‐fold increase in current density (4 A g^−1^), the full cell maintains a capacity of 42 mAh g^−1^, demonstrating its impressive rate capability. Upon changing back to the low current density of 0.02 A g^−1^, the discharge capacity delivered by the full cell returns to 120 mAh g^−1^, suggesting its satisfactory cycling stability. All the GCD curves are not straightly linear, which indicates a pseudocapacitive storage mechanism of the electrodes. These results are consistent with the observation in Figure 2. The cycling stability of the PTCDA//MVOH full cell is investigated at a current density of 4 A g^−1^. As shown in Figure 6D, the discharge capacity of the full cell decreases from the initial value of 58–36 mAh g^−1^ after 5000 cycles, with a capacity retention ratio of 62%, which is due to the gradual damage of Mg^2+^ insertion/extraction to the lamellar structure. Moreover, the Coulombic efficiency remains consistently ≈100%, demonstrating the high cycling stability of the PTCDA//MVOH full cell. Notably, the ultralong lifespan of 5000 cycles achieved by the PTCDA//MVOH full cell has surpassed those of the previously reported full MIBs (Table S6, Supporting Information). Moreover, the full cell in this study exhibits significantly higher capacity, exceptionally superior cycling life, and more high‐rate capability in comparison to other new kinds of metal ion batteries reported so far (Table S7, Supporting Information). The resulting full MIB cell shows a maximal energy density of 67 Wh kg^−1^ and a maximal power density of 2 kW kg^−1^; this energy density is remarkably high among aqueous full MIBs.^[^ ^37^, ^38^ ^]^

## 3 Conclusion

In this work, we prepared a novel lamellar material of Mg^2+^ pre‐intercalated hydrated V_10_O_24_·nH_2_O (denoted with MVOH) as MIB cathode. The pre‐intercalated Mg^2+^ cations and structural H_2_O molecules enlarge the interlayer spacing and stabilize the lamellar structure, which improves the Mg^2+^ migration kinetics and structure stability. The waltz‐like shuttle mechanism of H_2_O molecules coordinated with Mg^2+^ has been disclosed during the charging/discharging of the MVOH cathode: The Mg^2+^ ion migrates together with the coordinated H_2_O molecules (from IS to FS), while the H_2_O molecules adjust their orientations by rotating or even flipping to facilitate the zig‐zag migration of Mg^2+^, ensuring the stability of each step of the Mg^2+^ migration. Similarly, Mg^2+^ can also undergo symmetrical migration through the same mechanism (from FS to IS). After extraction of Mg^2+^ from MVOH, the Mg^2+^─H_2_O bond breaks, and the lattice H_2_O molecule creates empty chemical bonds, which allows it to rearrange and form H bonds in the interlayer of MVOH to stabilize the lamellar structure. With the shuttle function of lattice H_2_O molecules, the MVOH cathode exhibits a remarkably high discharge capacity of 350 mAh g^−1^ at a current density of 0.05 A g^−1^ and high‐rate capability (70 mAh g^−1^ at 4 A g^−1^). The aqueous full MIB cell based on MVOH cathode delivers an ultralong cycling life of 5000 cycles with a high energy density of 67 Wh kg^−1^. This work has revealed the sophisticated shuttle mechanism of lattice H_2_O molecules for Mg^2+^ migration, which is realized by the formation and cleavage of H bonds with VO_x_ layers. Due to the common existence of H bonds between H_2_O molecules and transition metal oxides, we anticipate that the waltz‐like Mg^2+^ shuttle mechanism of lattice H_2_O molecules is universal in transition metal oxide cathodes. Therefore, lattice H_2_O molecule is a favorable component for Mg^2+^ migration, which provides promises for advancing the development of MIBs for practical applications.

## Conflict of Interest

The authors declare no conflict of interest.

## Supporting information

Supporting Information

Supplemental Movie 1

## Data Availability

The data that support the findings of this study are available from the corresponding author upon reasonable request.
