# Supplementary material for: H2O‐Mg2+ Waltz‐Like Shuttle Enables High‐Capacity and Ultralong‐Life Magnesium‐Ion Batteries
Source: Adv Sci (Weinh). 2024 Apr 6;11(25):2401005. doi: 10.1002/advs.202401005 (PMC11220632; doi:10.1002/advs.202401005)
Supplement: Supplementary file 1 — Supporting Information [file ADVS-11-2401005-s001.pdf]

ADVANCED SCIENCE
Open Access

Supporting Information

for *Adv. Sci.*, DOI 10.1002/advs.202401005

H_2O-Mg^{2+} Waltz-Like Shuttle Enables High-Capacity and Ultralong-Life Magnesium-Ion Batteries

Xiu-Fen Ma, Bai-Qing Zhao, Hongyu Liu, Jing Tan, Hong-Yi Li, Xie Zhang*, Jiang Diao, Jili Yue, Guangsheng Huang, Jingfeng Wang and Fusheng Pan*

Supporting Information

H_2O-Mg^{2+} waltz-like shuttle enables high-capacity and ultralong-life magnesium-ion batteries

Xiu-Fen Ma#, Bai-Qing Zhao#, Hongyu Liu, Jing Tan, Hong-Yi Li, Xie Zhang*, Jiang Diao, Jili Yue, Guangsheng Huang, Jingfeng Wang, and Fusheng Pan*

#X.-F. Ma and B.-Q. Zhao contributed equally to this work

X.-F. Ma, H. Liu, J. Tan, H-Y Li, J. Diao, J. Yue, G. Huang, J. Wang, and F. Pan
National Innovation Center for Industry-Education Integration of Energy Storage Technology
College of Materials Science and Engineering
Chongqing University
Chongqing 400044, China
*E-mail: hongyi.li@cqu.edu.cn

B.-Q. Zhao
Materials and Energy Division
Beijing Computational Science Research Center
Beijing 100193, China

X. Zhang
School of Materials Science and Engineering
Northwestern Polytechnical University
Xi'an 710072, China
*E-mail: xie.zhang@nwpu.edu.cn

H-Y Li, J. Diao, J. Yue, G. Huang, J. Wang, and F. Pan
National Engineering Research Center for Magnesium Alloys
Chongqing University
Chongqing 400044, China

F. Pan

National Key Laboratory of Advanced Casting Technologies

Chongqing University

Chongqing 400044, China

Contents

Figure S1. XPS survey spectrum of MVOH nanoflowers……S1

Figure S2. TEM image of the MVOH……S2

Figure S3. Scanning transmission electron microscopy (STEM) with EDS mapping of Mg, V, O elements……S2

Figure S4. Raman spectrum of MVOH nanoflowers……S3

Figure S5. FT-IR spectrum of MVOH nanoflowers……S4

Figure S6. (a) N_2 adsorption/desorption isotherm of MVOH nanoflowers and the straight line fitted with BET theory (inset); (b) the BJH pore-size distribution plots of MVOH……S5

Figure S7. The scheme of structural construction and optimization for MVOH. The orthorhombic V_2O_5 structure was optimized and utilized as the basis for constructing the MVOH configuration……S6

Figure S8. Cyclic voltammogram of MVOH cathode between -0.7 and 1.0 V (vs. SCE) at a scan rate of 1 mV s^{-1}……S6

Figure S9. Cycling stability test results of the MVOH cathode at 3 A g^{-1} in the three-electrode system……S7

Figure S10. Kinetics analysis of the VOH cathode……S8

Figure S11. (a) EIS spectrum; (b) The linear regression of Z' plotting against $\omega^{-1/2}$……S9

Figure S12. Ex situ HRTEM images in MVOH cathode at pristine, discharged to -0.7 V, and charged to 1.0 V states……S9

Figure S13. The structural change of MVOH during the H_2O-Mg^{2+} waltz-like shuttle……S10

Figure S14. Depth profiles of O^+, Mg^+ and $CF_3SO_3^-$ of fully discahged and charged electrode……S10

Figure S15. The volume change of MVOH before and after Mg^{2+} insertion……S11

Figure S16. Schematic illustration of full cell with MVOH cathode and PTCDA anode……S11

Table S1. ICP-OES results of Mg and V elements in MVOH nanoflowers……S12

Table S2. Atom fraction results of STEM-EDS in MVOH powder sample……S12

Table S3. Electrochemical performance comparison between MVOH cathode and reported cathode materials……S13

Table S4. Diffusion coefficient comparison of Mg^{2+} in MVOH cathode and reported cathode materials……S14

Table S5. Migration barrier energy comparison between MVOH cathode and other vanadium oxides..... S14

Table S6. Electrochemical performance comparison between our PTCDA//MVOH full cell and reported aqueous/non-aqueous Mg ion batteries……S15

Table S7. Electrochemical performance comparison between our PTCDA//MVOH and other reported metal-ion full batteries……S16

Experimental Section……S17

Supplementary references……S19

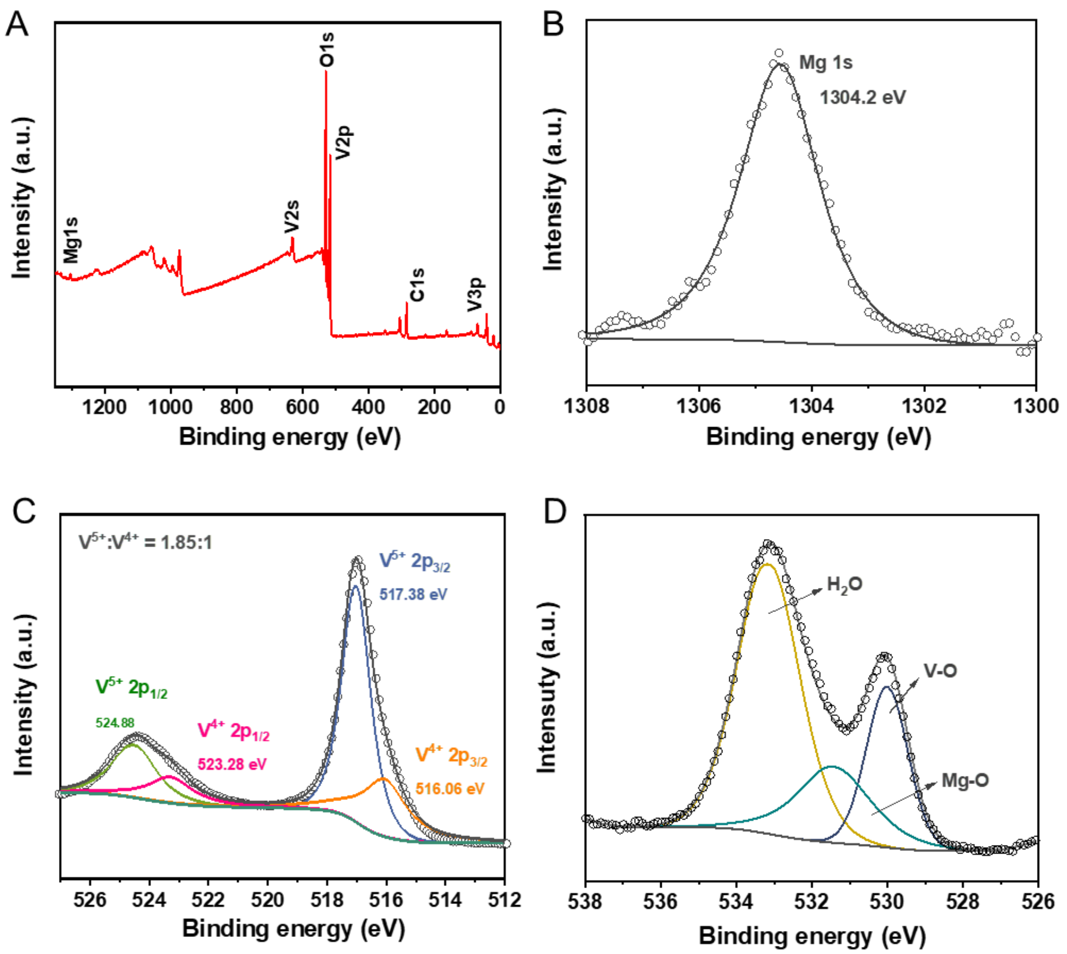

Figure S1. XPS survey spectrum of MVOH nanoflowers (a), XPS spectra of Mg 1s (b), V 2p (c) and O 1s (d) levels in MVOH nanoflowers.

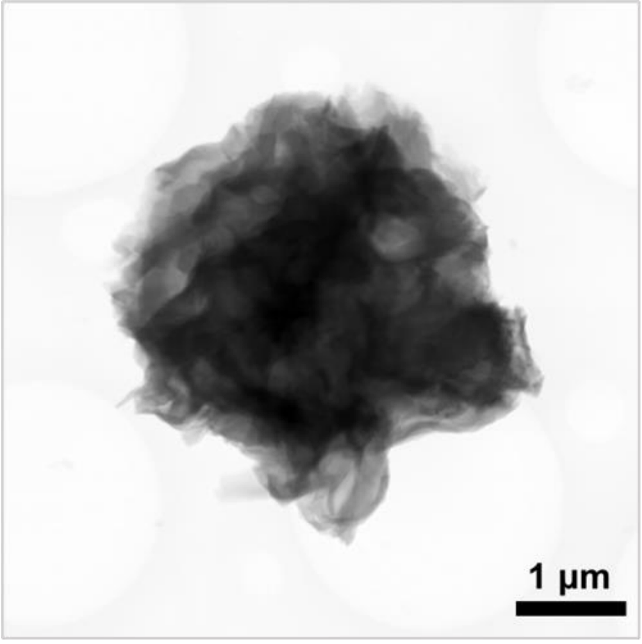

Figure S2. TEM image of the MVOH.

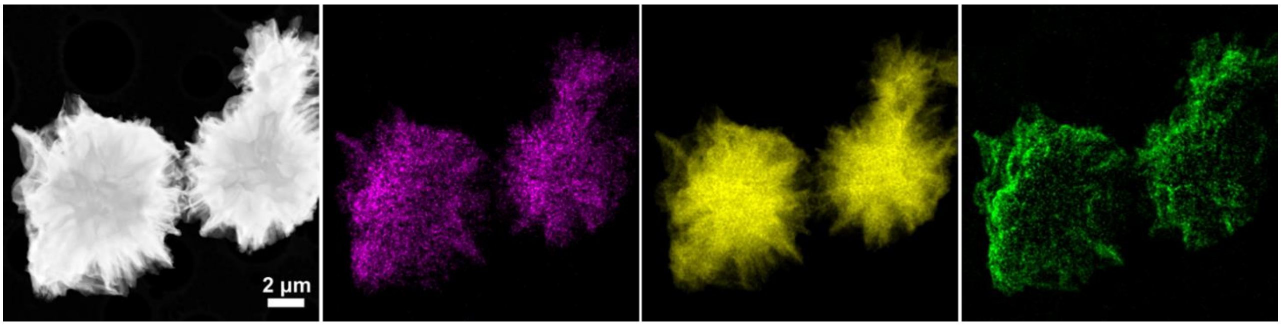

Figure S3. Scanning transmission electron microscopy (STEM) with EDS mapping of Mg, V, O elements.

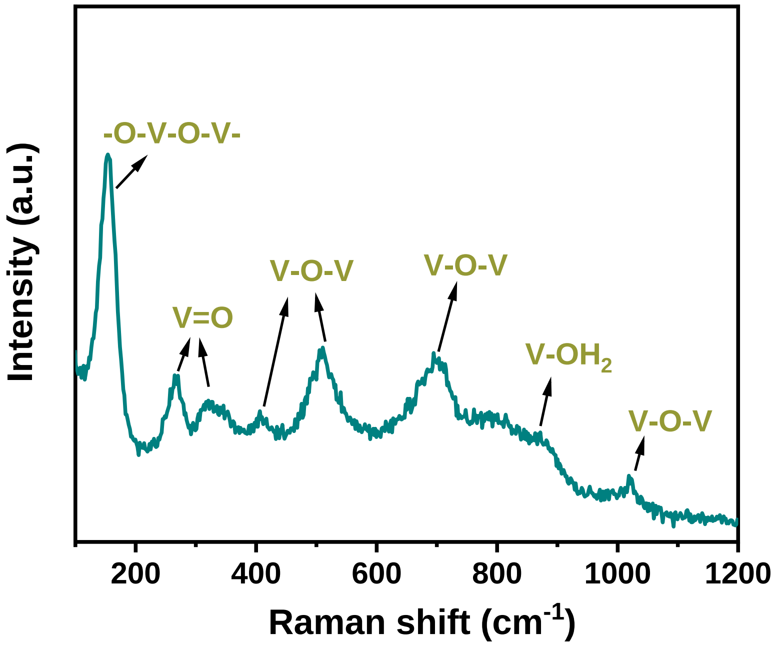

Figure S4. Raman spectrum of MVOH nanoflowers.

The Raman spectrum of MVOH suggests the existence of V-O-V and V=O bonds, confirming the crystal structure of MVOH shown in Figure S4. The strong signal at 155 cm^{-1} reflects the -O-V-O-V- chain, which is caused by the relative motions of V-O bonds in the VO_4 tetrahedra or VO_6 octahedra, and this is the exclusive vibration mode of the layered structure.[S1] The bands at 267 and 319 cm^{-1} are attributed to the bending vibration mode of V=O bonds.[S2-S3] The adsorption bands at 404, 512, 698 and 1018 cm^{-1} are due to the stretching vibration of V-O-V bonds.[S4] The weak peak at 871 cm^{-1} is related to the stretching vibration of V-OH_2 bonds, which indicates that H_2O molecules are inserted into the interlayer spacing and coordinate with V atoms in the V-O skeleton layer.[S5]

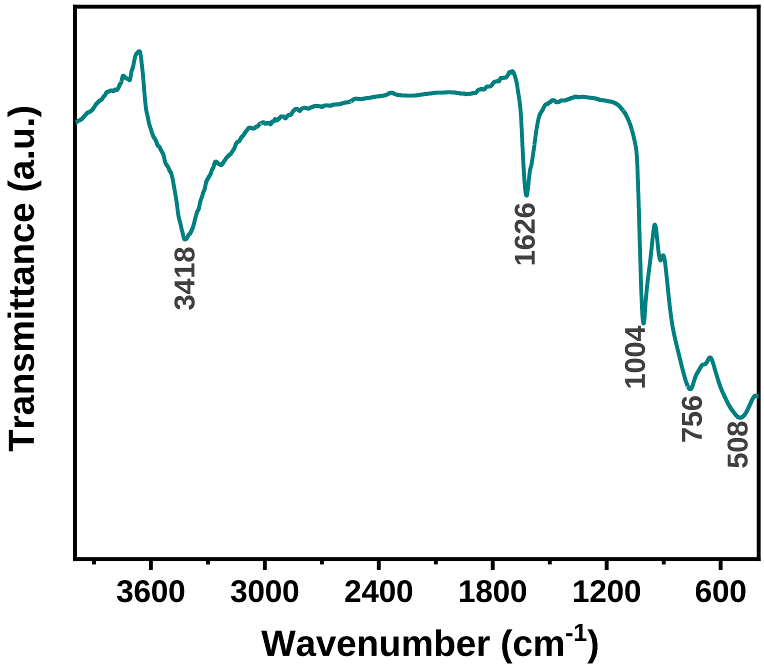

Figure S5. FT-IR spectrum of MVOH nanoflowers.

In the Fourier-transformed infrared (FT-IR) spectra (Figure S5), the absorption bands at 3418 cm^{-1} and 1626 cm^{-1} further indicate the existence of H_2O molecules in MVOH, which are attributed to the O-H stretching vibration and the H-O-H bending vibration, respectively. The absorption band at 1004 cm^{-1} originates from the V=O vibration. The signal at 756 cm^{-1} is attributed to the asymmetric stretching vibration of V-O-V bonds, while the signal at 508 cm^{-1} is due to the shear vibration of V-O-V bonds.[S6-S7]

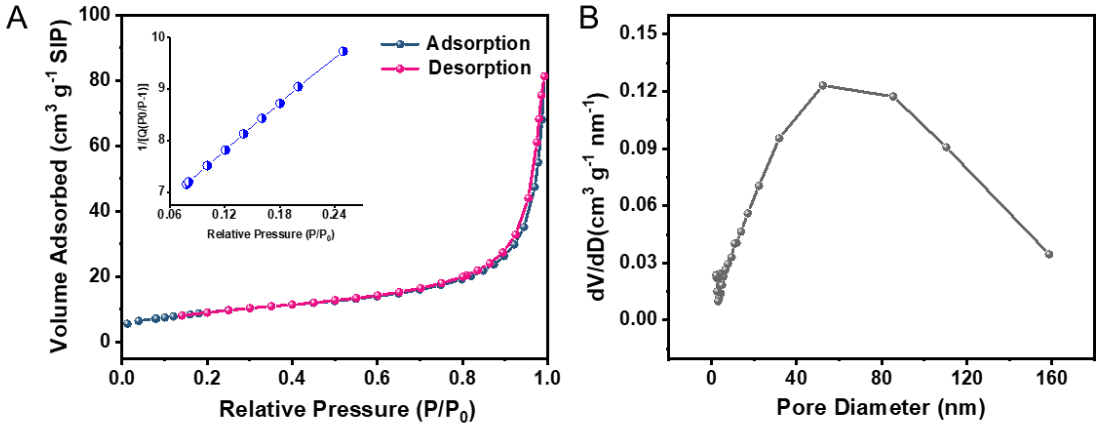

Figure S6. (a) N_2 adsorption/desorption isotherm of MVOH nanoflowers and the straight line fitted with BET theory (inset); (b) the BJH pore-size distribution plots of MVOH.

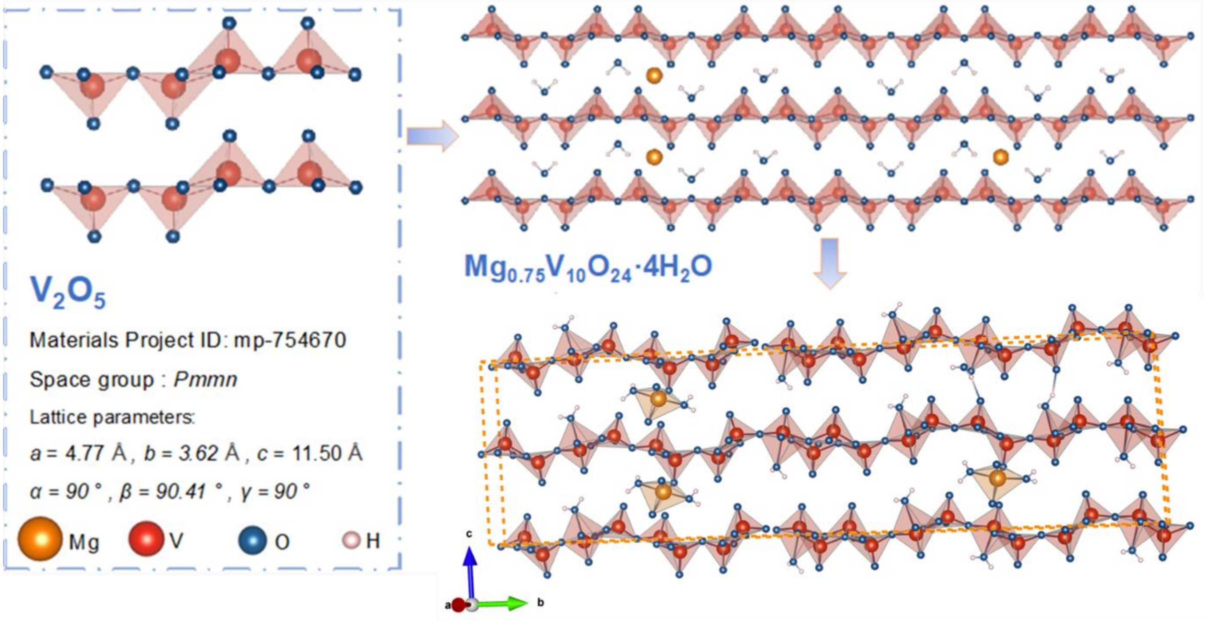

Figure S7. The scheme of structural construction and optimization for MVOH. The orthorhombic V_2O_5 structure was optimized and utilized as the basis for constructing the MVOH configuration.

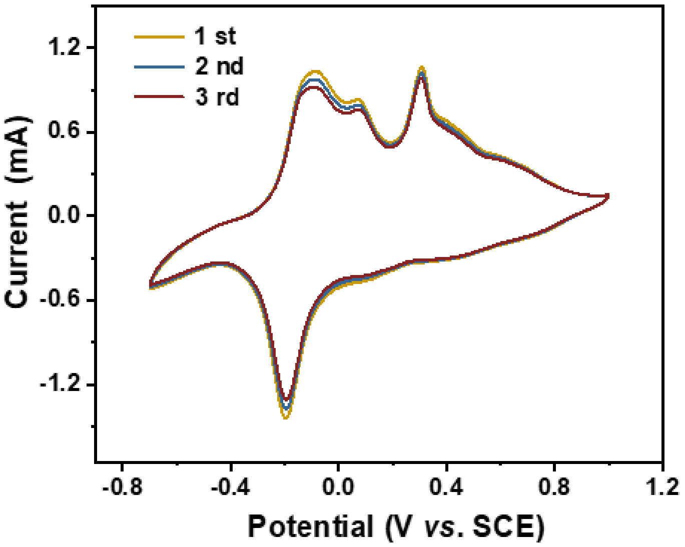

Figure S8. Cyclic voltammogram of MVOH cathode between -0.7 and 1.0 vs. SCE at a scan rate of 1 mV s^{-1}**.**

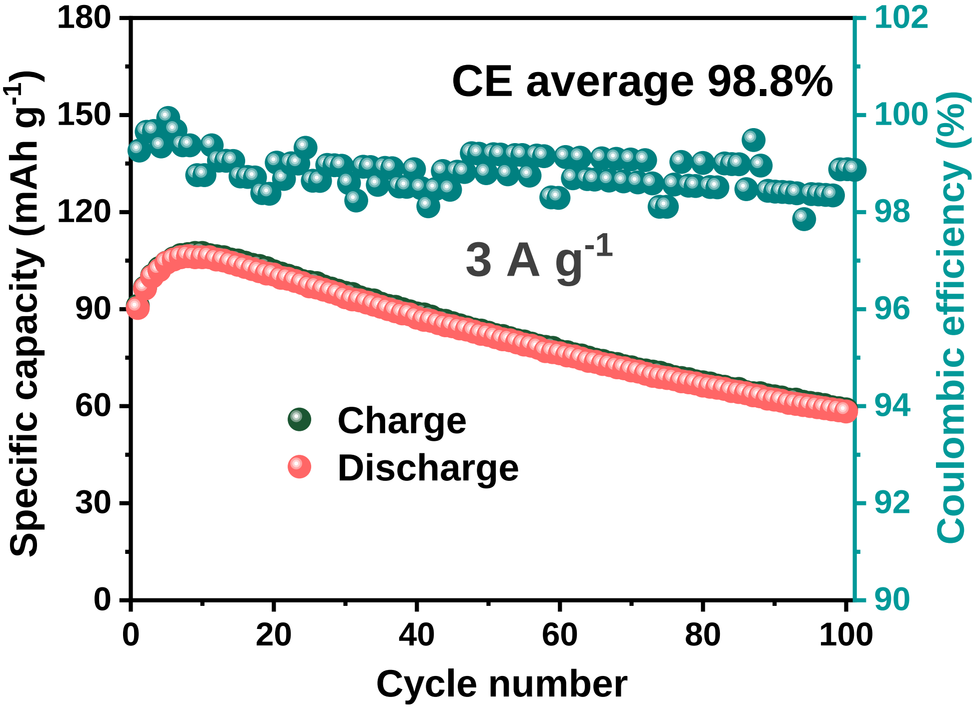

Figure S9. Cycling stability test results of the MVOH cathode at 3 A g^{-1} in the three-electrode system.

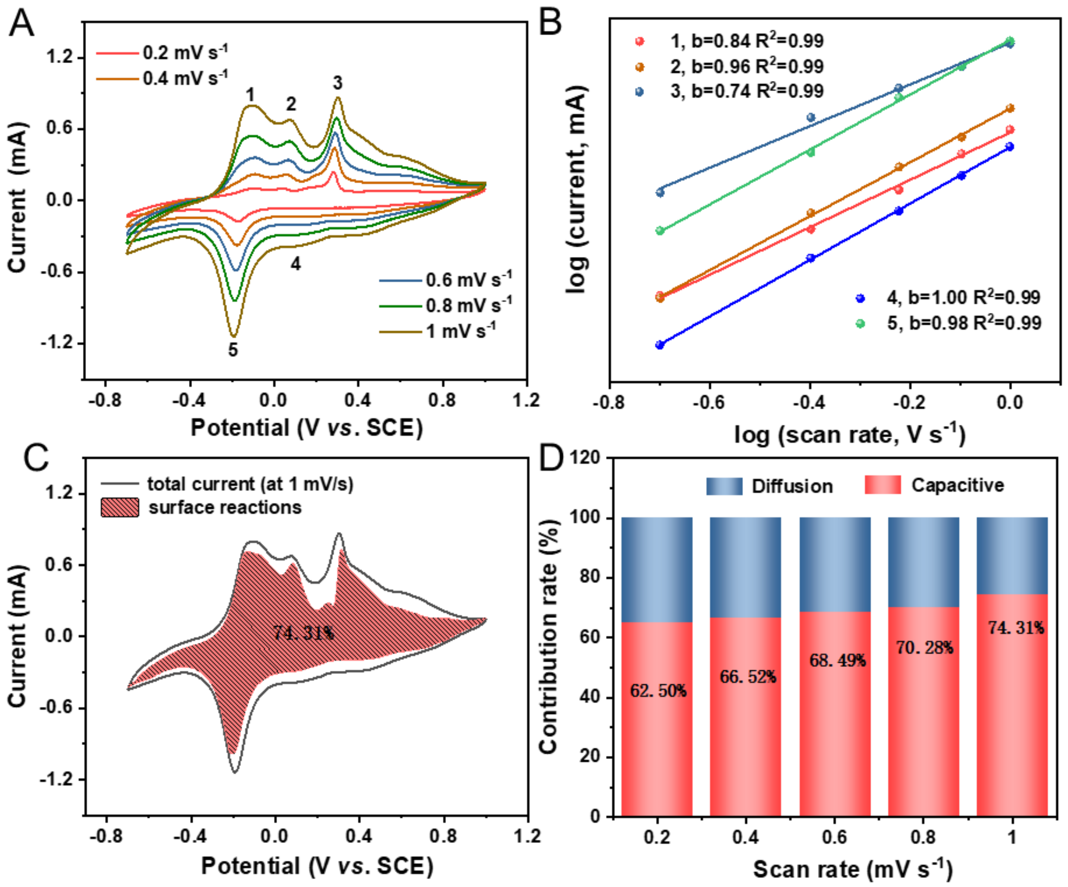

Figure S10. Kinetics analysis of the VOH cathode: (a) CV curves at different sweep rates; (b) The relationship of peak current and scan rate; (c) Capacitive (surface reactions) contribution to charge storage at 1 mV s^{-1}; (d) Normalized contribution ratio of capacitive contribution to the total capacity at various scan rates.

From the peak currents of Peak 1-5 in Figure S10a, the corresponding b values obtained by regression are 0.84, 0.96, 0.74, 1.00 and 0.86, respectively (Figure S10b). Furthermore, the pseudocapacitance (k_1v) and diffusion ($k_2v^{1/2}$) dominated capacities of the MVOH cathode were quantitatively evaluated by utilizing the equation $i(V) = k_1v + k_2v^{1/2}$, where $i(V)$ represents the current at a specific voltage (V).[S8] As the scan rate varies from 0.2 mV s^{-1} to 1.0 mV s^{-1}, the calculated contribution ratio of pseudocapacitive capacity increases from 62.50% to 74.31% (Figure S10d).

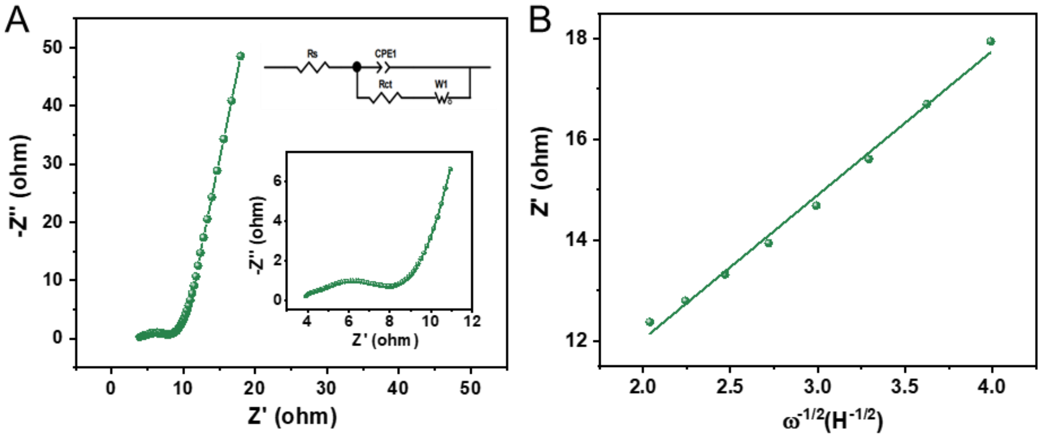

Figure S11. (a) EIS spectrum; (b) The linear regression of Z’ plotting against $\omega^{-1/2}$.

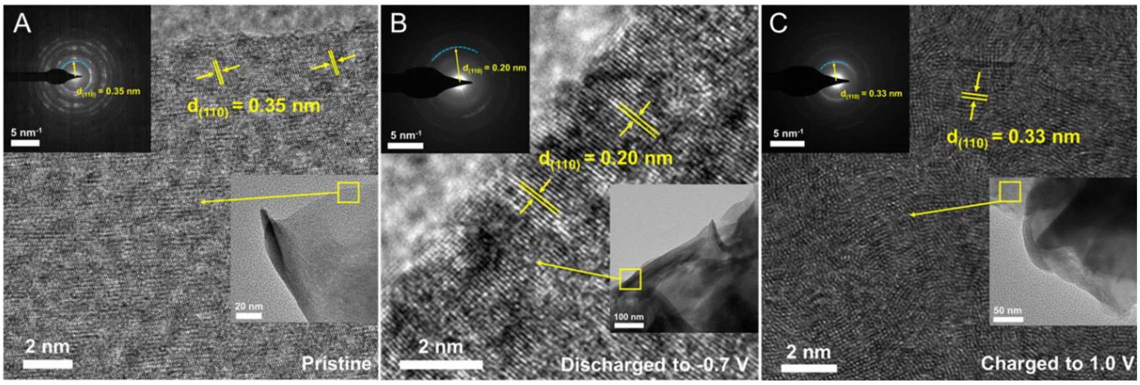

Figure S12. Ex situ HRTEM images in MVOH cathode at pristine, discharged to -0.7 V, and charged to 1.0 V states.

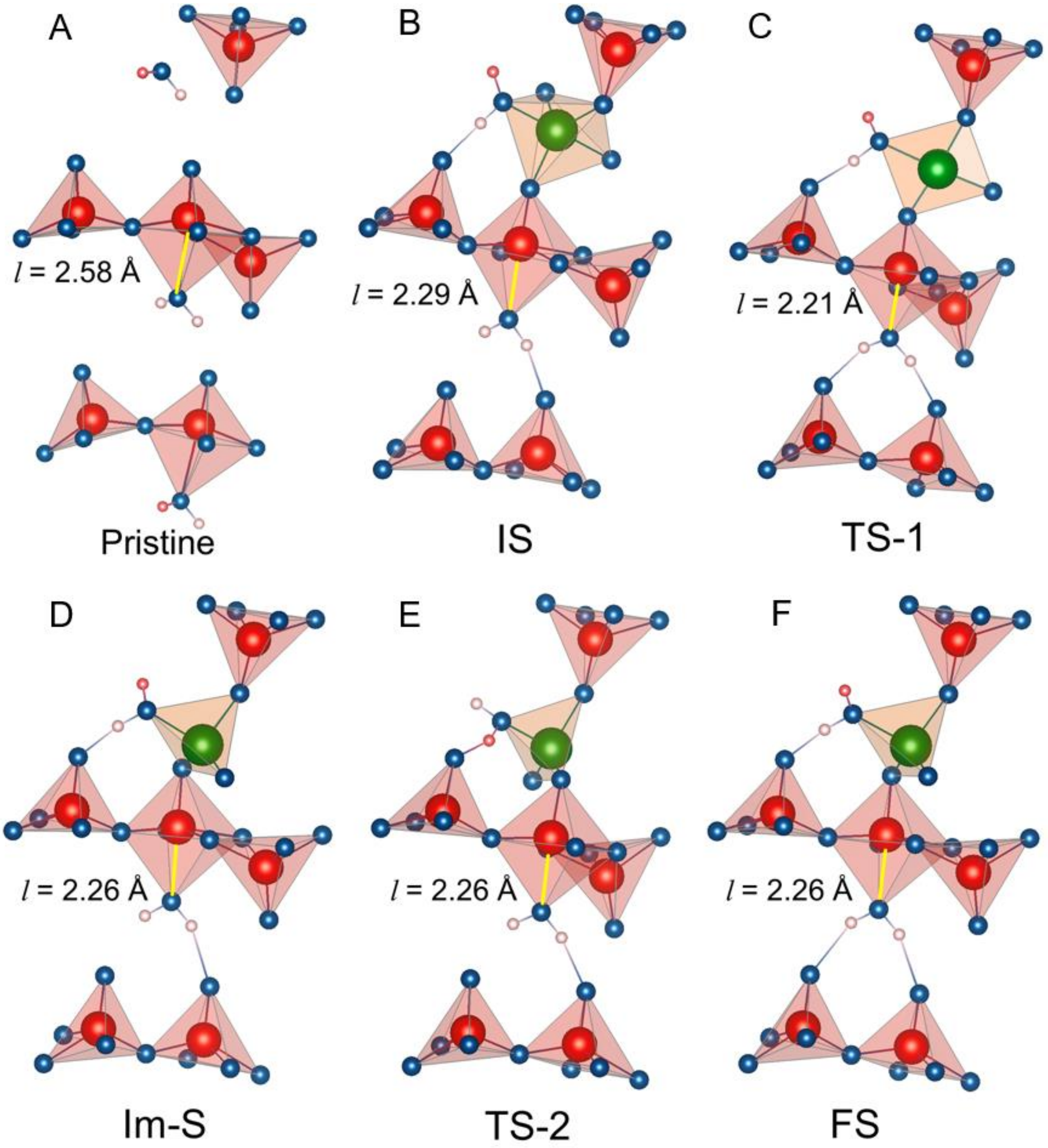

Figure S13. The structural change of MVOH during the H_2O-Mg^{2+} waltz-like shuttle.

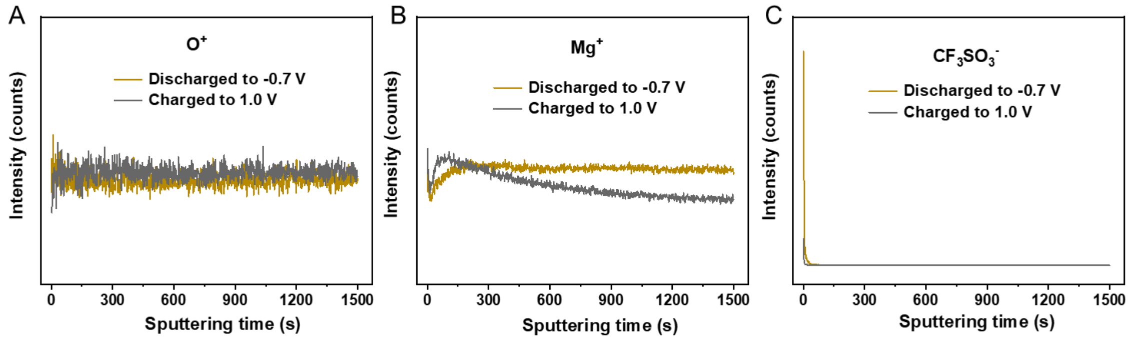

Figure S14. Depth profiles of O^+, Mg^+ and $CF_3SO_3^-$ of fully discahged and charged electrode.

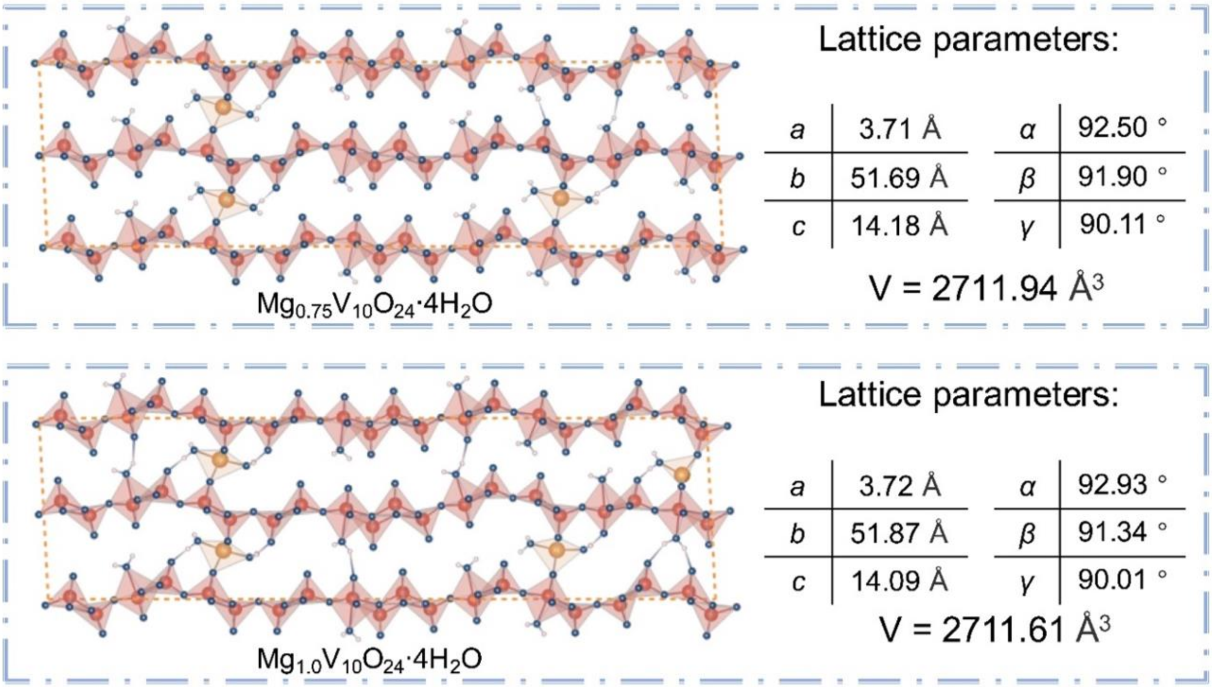

Figure S15. The volume change of MVOH before and after Mg^{2+} insertion

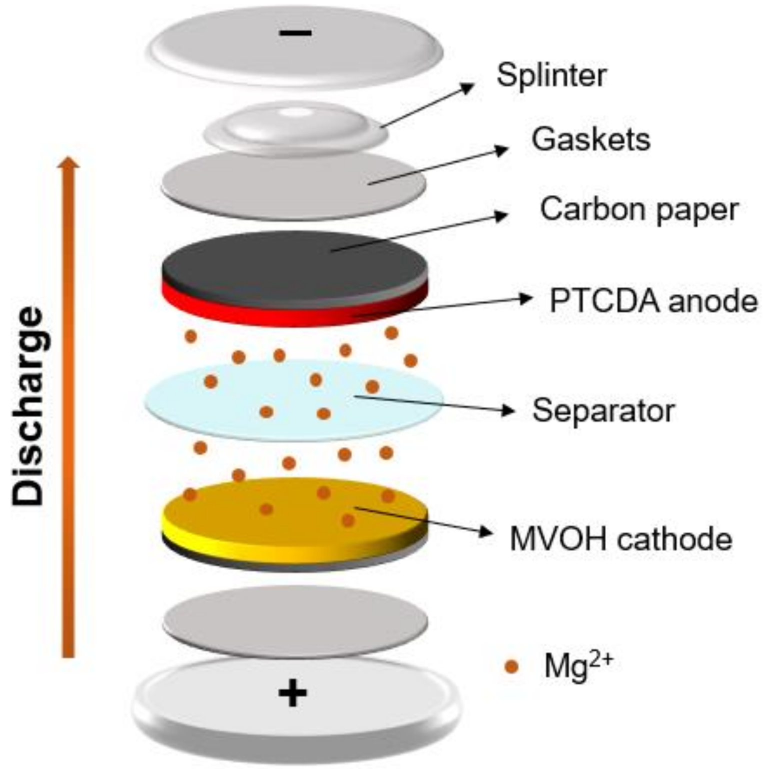

Figure S16. Schematic illustration of full cell with MVOH cathode and PTCDA anode.

Table S1

ICP-OES results of Mg and V elements in MVOH nanoflowers.

Elements	C_0(mg/L)	Mg:V (atomic ratio)
Mg/V	0.2436/6.894	0.75:10.0

Table S2

Atom fraction results of STEM-EDS in MVOH powder sample.

Elements	Atom Fraction (%)	Atom Error (%)
O	71.37	8.13
Mg	2.01	0.49
V	26.62	4.15

Table S3

Electrochemical performance comparison between MVOH cathode and reported cathode materials.

Cathode	Electrolyte (solvent: water)	Operating voltage (V)	capacity (mAh g^{-1}@A g^{-1})	References
**MVOH**	**2 M $Mg(CF_3SO_3)_2$**	**1.7**	**350@0.05**	**This work**
$MgFeMnO_4$	0.5 M $MgCl_2$	1.9	136.5@0.05	Chem. Eng. J., 2020, 392,123652
Birnessite $Mg_{0.15}MnO_2{\cdot}0.9H_2O$/carbon cloth composite	2 M $Mg(ClO_4)_2$	1.6	150@2C	Chem. Mater., 2016, 28, 534-542
$Li_3V_2(PO_4)_3$	4 M Mg(TFSI)$_2$	1.2	115.1@0.1	ACS Cent. Sci., 2017, 3, 1121-1128
Polyaniline	4.5 M $Mg(NO_3)_2$	0.9	100@1C	Adv. Funct. Mater., 2021, 31, 2107523
$K_2CuFe(CN)_6$	1 M $Mg(CH_3COO)_2$	0.9	52@0.1	Angew. Chem. Int. Ed., 2023, e202308961
Prussian blue type nickel hexacyanoferrate	1 M $MgSO_4$	0.95	65@0.1	ACS Energy letters, 2017, 2: 1115.
Mg-OMS-1/Graphene	0.5 M $Mg(NO_3)_2$	1.45	200@0.02	ACS Sustainable Chem. Eng., 2019, 7, 6113-6121.
δ-MnO_2	1 M $MgCl_2$	1.7	252.1@0.05	J. Energy Chem., 2022, 68, 572-579
$Mg_{0.58}MnO_2{\cdot}0.42H_2O$	0.5 M $MgCl_2$	1.8	169.3@0.05	J. Magnes. Alloy, 2023, 11, 840-850
$K_2CuFe(CN)_6$	Hydrated eutetic electrolyte	1.2	61.2@0.5C	Energy Environ. Sci., 2022, 15, 1282
$MgMn_2O_4$	0.5 M $Mg(NO_3)_2$	1.3	185.1@0.1	Chem. Eng. J., 2022, 435, 134997.

Table S4

Diffusion coefficient comparison of Mg^{2+} in MVOH cathode and reported cathode materials.

Cathode	D_{Mg} (cm^2 s^{-1})	References
**MVOH**	**1.08×10^{-9}**	**This work**
$FeVO_4$	4.61×10^{-11}	Chem. Eur. J., 2017, 23, 17118-17126
$FeVO_4$/C	1.53×10^{-10}	Chem. Eur. J., 2017, 23, 17118-17126
$Mg_{1.1}Mn_6O_{12}\cdot4.5H_2O$	6.65×10^{-12}	J. Power Source, 2017, 338, 136-144
Mg-OMS-1/Graphene	1.15×10^{-14}	ACS Sustainable Chem. Eng., 2019, 7, 6113-6121.
Ni_x-Mg-1	5.13×10^{-16}	Inorg. Chem. Front., 2020,7, 2168-2177
$MgFe_xMn_{2-x}O_4$	3.26×10^{-13}	Chem. Eng. J., 2019, 123652
$Mg_{0.58}MnO_2\cdot0\cdot56H_2O$	5.40×10^{-10}	J. Magnes. Alloy, 2023, 11, 840-850

Table S5

Migration barrier energy comparison between MVOH cathode and other vanadium oxides.

Materials	Migration barrier energy	References
MVOH	0.54	This work
α-V_2O_5	1.15	Chem. Mater., 2016, 28, 5611-5620
ζ-V_2O_5	0.86	Chem. Mater., 2016, 28, 5611-5620
δ-V_2O_5	1.38	Phys. Chem. Chem. Phys., 2014, 16, 18578-18585
Single-layered V_2O_5	1.40	Phys. Chem. Chem. Phys., 2013, 15, 8705-8709
bulk V_2O_5	1.36	Phys. Chem. Chem. Phys., 2013, 15, 8705-8709
ε-$Mg_{0.5}V_2O_5$	1.06	J. Phys. Chem. C, 2018, 122, 1513-1521
δ-MgV_2O_5	0.68	J. Phys. Chem. C, 2018, 122, 1513-1521

Table S6

Electrochemical performance comparison between our PTCDA//MVOH full cell and reported aqueous/non-aqueous Mg ion batteries.

Cell Description	Electrolyte Solution	Highest Capacity (mAh/g)	Highest Rate	Cycle Life/Capacity retention rate(%)	References
**PTCDA//MVOH**	**2 M $Mg(CF_3SO_3)_2$ in H2O+PEG (1+1)**	**133**	**4 A g^{-1}**	**5000/62**	**This work**
Prussian blue type nickel hexacyanoferrate//polyimide	1 M $MgSO_4$ in water	37	10 A g^{-1}	5000/60	ACS Energy Lett. 2017, 2, 1115-1121
PTCDA// $K_2CuFe(CN)_6$	Hydrated eutetic electrolyte in water	38.1	20C	1000/65.3	Energy Environ. Sci., 2022, 15, 1282
PANI//PDI-EDA	4.5 M $Mg(NO_3)_2$ in water	45	200C	6000/67.5	Adv. Funct. Mater. 2021, 31, 2107523
Mg// $K_2CuFe(CN)_6$	$MgCl_2{\cdot}6H_2O$ water in salt	47	5 A g^{-1}	700/57	ACS Energy Lett. 2022, 7, 2657−2666
VO_2(B)//CuHCF	1 M $Mg(CH_3COO)_2$ in water	~150	0.5 A g^{-1}	500/70.2	Angew. Chem. Int. Ed., 2023, e202308961
PPMDA@MCNTs//MgxLi$V_2(PO_4)_3$	4 M $Mg(TFSI)_2$ in water	50	60C	1000/86.8	ACS Cent. Sci., 2017, 3, 1121-1128
$FeVO_4$/C// Mg-OMS-1/Graphene	1 M $MgSO_4$ in water	78.2	0.5 A g^{-1}	100/97.7	Chem. Eur. J. 2017, 23, 17118-17126
Carbon molecular sieves//Mg-OMS-1/Graphene	0.5 M $Mg(NO_3)_2$ in water	70.6	0.5 A g^{-1}	300/98.6	ACS Sustainable Chem. Eng., 2019, 7, 6113-6121.
Mg//Mo_6S_8	0.125 M $Mg(CF_3SO_3)_2$ in THF:PP_{14}TFSI (16:1)	86	0.3C	100/~78	Nano Energy 2023, 109, 108257
Mg//Mo_6S_8	0.5 M $Mg(TFSI)_2$ in DME	110	-	400/84.8	ACS Nano 2023, 17, 8980−8991
Mg//CuS	Magnesium lithium chloride comples in THF+BTFE (1+1)	260	5C	800/61.5	Adv. Energy Mater. 2022, 12, 2202602
Mg@3D-Mg affinity controlled architecture// Mo_6S_8	APC	110	-	300/87.6	Energy Storage Mater. 2023,59, 102762
Mg//Mo_6S_8	0.5 M $Mg(TFSI)_2$ in DME	90	2C	1000/87.7	Adv. Funct. Mater. 2022, 32, 2208735

Table S7

Electrochemical performance comparison between our PTCDA//MVOH and other reported metal-ion full batteries.

Cell Description	Highest capacity (mAh/g)	Highest Rate	Cycle Life/Capacity retention rate(%)	References
**PTCDA//MVOH**	**133**	**4 A g^{-1}**	**5000/62**	**This work**
SnPx@C/reduced graphene oxide //$Na_3V_2(PO_4)_2F_3$ (Na^+ , Organic electrolyte)	98	0.8 A g^{-1}	200/78	Adv. Funct. Mater. 2020, 2003086
Graphite//$KVPO_4F$ (K^+ , Organic electrolyte)	65	0.05 A g^{-1}	200/87.2	Adv. Funct. Mater. 2023, 2305829
Hard carbon//$Na_{3.05}V_{1.03}Fe_{0.97}(PO_4)_3$ (Na^+ , Organic electrolyte)	102.5	20C	500/92	Adv. Sci. 2023, 10, 2301308
Graphite//polytriphenylamine (Dual ion , Organic electrolyte)	44	0.3 A g^{-1}	500/75.5	ACS Energy Lett. 2017, 2, 1614-1620
VO_2//$Na_3(VO)_2(PO_4)_2F$ (Na^+, Organic electrolyte)	90	20C	-	Adv. Energy Mater. 2018, 1800058
Hard carbon//Protonated polyaniline (Na^+, Organic electrolyte)	97	5 A g^{-1}	2000/-	Adv. Funct. Mater. 2021, 2107830
Sb-CNT//$Na_3V_2(PO_4)_2O_2F$ (Na^+ , Organic electrolyte)	120	20C	-	Adv. Mater. 2017, 1701968
MCMB//$Na_3V_2(PO_4)_2O_2F$ (Dual-ion, Organic electrolyte)	＜120	2.6 A g^{-1}	2000/86.3	Adv. Energy Mater. 2018, 8, 1702504
Nb_2O_{5-x}@rGO//$K_{0.5}V_2O_5$ (K^+, Organic electrolyte)	58.4	0.2 A g^{-1}	270/81.3	Small 2019, 15, 190127
$Na_2Ti_3O_7$//$VOPO_4$ (Na^+, Organic electrolyte)	114	5C	100/92.4	Energy Environ. Sci, 2016, 9, 3399-3405
P2/P3-$Na_{0.7}Li_{0.06}Mg_{0.06}Ni_{0.22}Mn_{0.67}O_2$ (Na^+, Organic electrolyte)	97	5C	50/67.7	Nano Energy, 2019, 55, 143-150
Hard carbon// $Na[Cu_{1/9}Ni_{2/9}Fe_{1/3}Mn_{1/3}]O_2$ (Na^+, Organic electrolyte)	~100	2C	1200/71	Energy Storage Mater. 2019, 18, 269–279
Graphite//polytriphenylamine (K^+, Organic electrolyte)	44	0.3 A g^{-1}	500/75.5	ACS Energy Lett., 2017, 2, 1614−1620
Pb//$Na_3V_2(PO_4)_3$-C (Na^+, Organic electrolyte)	104	5C	300/~34	Nano Energy, 2022, 95, 107010
Tin phosphide//$Na_3V_2(PO_4)_2F_3$, (Na^+, Organic electrolyte)	100	0.2 A g^{-1}	-	Adv. Funct. Mater. 2020, 2003086

Experimental Section

Materials: Vanadium (V) oxide V_2O_5 (≥99.5%), 30 wt% hydrogen peroxide H_2O_2, Magnesium nitrate hexa hydrate $Mg(NO_3)_2{\cdot}6H_2O$ (≥99.0%), magnesium trifluoromethanesulfonate $Mg(CF_3SO_3)_2$ (≥99.0%), polyethylene glycol PEG 400 (≥99.0%), Perylene-3,4,9,10-tetracarboxylic anhydride (PTCDA, 99.9%). All reagents were used as received without additional purification.

Materials Synthesis: The synthesis process for $Mg_{0.75}V_{10}O_{24}{\cdot}4H_2O$ nanoflowers typically involves the following steps: Vanadium pentoxide (V_2O_5, 0.25 g) was added to 3 mL of hydrogen peroxide (H_2O_2) with a volume fraction of 30%, and at the end of the exothermic reaction a clear reddish-brown solution was displayed and 50 mL of deionized water added. Subsequently, magnesium nitrate hexahydrate ($Mg(NO_3)_2{\cdot}6H_2O$, 1.8 g) was added to the above solution and placed on a heated plate at 300 °C for 6 h. Then, the precipitation product was washed with anhydrous ethanol and deionized water for three times, and dried in a vacuum drying oven at 70 ℃ for 12 h. Finally, the brown powder sample of MVOH was collected.

Materials Characterization: The powder X-ray diffraction (XRD) of the as-prepared MVOH were collected on a Rigaku D/Max 2500 PC using Cu Kα irradiation (λ=0.1514178 nm). Scanning electron microscopy (SEM) was performed using Quattro S to observe the microstructure of the prepared samples. Transmission electron microscopy (TEM) and high-resolution TEM (HRTEM) analyses were conducted using Talos F200S to obtain detailed information about the nanostructure and crystal lattice of the MVOH. The sample composition quantitatively was tested using ICP-OES (PerkinElmer 8300). Thermogravimetry-differential thermal analysis (TG-DSC, Netzsch STA 449C) was used to determine the molecular formular of MVOH nanoflowers at a heating rate of 15 °C min^{-1}. The Raman spectra were measured using a LabRAM HR Evolution system that had a 532 nm laser and confocal objective. For the Fourier transform infrared spectra (FT-IR), a Nicolet iS50 was employed, covering a wavelength range of 400~4000 cm^{-1} and a resolution of 0.09 cm^{-1}. X-ray photoelectron spectroscopy (XPS, ESCALAB 250Xi) was used to analyze the oxidation and reduction states of Mg, V and O elements. The N_2 adsorption/desorption isotherms were determined using Brunauer-Emmett-Teller (BET) tests on an automatic specific surface analyzer. The pore-size distribution was obtained using the Barrett-Joyner-Halenda (BJH) method.

Electrochemical Measurements: All electrochemical tests are performed at room temperature. The electrochemical characterization of MVOH nanoflowers was conducted using a conventional standard three-electrode electrochemical cell. Platinum foil (with a thickness of ca. 0.1 mm) was used as the counter electrode, while a saturated calomel electrode (SCE) served

as the reference electrode. The working electrode were fabricated by mixing the active material (MVOH nanoflowers), acetylene black and polyvinylidene fluoride (PVDF) with the weight ratio of 7:2:1 in the N-methyl-2-pyrrolidone (NMP) solvent to form a slurry. Once thoroughly mixed, the slurry was applied onto carbon fiber cloths (1 × 1.5 cm^2, a thickness of ca. 0.3 mm) and subsequently dried in a vacuum oven at 80°C overnight to form the electrodes. Each electrode had a mass loading of approximately 2.0~2.5 mg cm^{-2}, containing the active material. The electrolyte employed in the study consisted of a 2 mol L^{-1} aqueous solution of $Mg(CF_3SO_3)_2$. This electrolyte was prepared by combining deionized water with analytical-grade magnesium salt regents. To characterize the electrochemical properties of the system, a Chenhua CHI660E electrochemical workstation was utilized. This instrument allowed for the measurement of key parameters such as cyclic voltammetry (CV), galvanostatic charging and discharging (GCD), and electrochemical impedance spectroscopy (EIS) curves. The impedance measurements were conducted over a frequency range of 0.01 to 10^5 Hz. The diffusion coefficient (D_{Mg}) of Mg^{2+} ion can be calculated using Equation (S1) and (S2),

$$D_{Mg} = 0.5(RT/n^2F^2AC_{Mg}A_w)^2 \quad \text{(S1)}$$

$$Z' = R_s + R_{ct} + A_w\omega^{-1/2} \quad \text{(S2)}$$

In the given equation, R represents the gas constant, T denotes the room temperature, n represents the number of electrons transferred (in this case, n=1), F represents the Faraday constant, A represents the surface area of the electrode, C_{Mg} represents the concentration of Mg^{2+}, and A_w represents the slope of Z′ plotted against $\omega^{-1/2}$ (as shown in Figure 2i). Here, Z′ represents the real impedance, and ω represents the angular frequency.

The assembled PTCDA//MVOH full cells in atmospheric air were at the mass ratio of active materials of ~3 (PTCDA, 3.6 mg cm^{-2}) : 1 (MVOH, 1.2 mg cm^{-2}). A thinner carbon paper of 0.1 mm as current collector. The electrolyte utilized in the experiment consisted of a 2 mol L^{-1} $Mg(CF_3SO_3)_2$ solution prepared by mixing deionized water and polyethylene glycol in a 1:1 volume ratio. The amount of electrolyte in each full cell is 100 μL. As for the separator, a glass fiber separator of Grade GF/A manufactured by Whatman was employed. The electrochemical performance of full cells was assessed through cyclic voltammetry (CV) and galvanostatic charge-discharge (GCD) measurements. CV tests were carried out using the CHI 660E electrochemical workstation from Chenhua. The galvanostatic charge-discharge tests were performed using a multichannel battery testing machine (CT4008A, Neware) at various current densities within the potential range of 0 to 1.7 V. The energy density (E, Wh kg^{-1}) and power density (P, W kg^{-1}) of the full cell were determined by analyzing the galvanostatic charge/discharge curves using the following equations:

$$E = \int_{t1}^{t2} UIdt \quad (S3)$$

$$p = \frac{E}{t} \quad (S4)$$

where t_1 and t_2 represent the start and end times (in seconds) of the discharge, respectively; Δt is the difference between t_2 and t_1 (in seconds); I is the discharge current (in Amperes); U is the voltage range (in Volts). In this case, the specific capacity, energy density, and power density are determined by considering the mass of the cathode as the reference parameter for calculations.

Computational Methods: Density functional theory (DFT) calculations were performed using the Vienna *Ab-initio* Simulation Package (VASP). [S9] To optimize the crystal structure, the projector augmented wave (PAW) pseudopotentials with the Perdew-Burke-Ernzerhof (PBE) exchange-correlation functional were employed. [S10] Additionally, to account for the strong electron correlation effects inherent to transition metal V elements, the DFT+*U* scheme was used for our calculations. [S11] The *U* parameter of 3.25 eV was applied to the vanadium *d* states based on careful tests in the literature.[S12] A Γ-centered Monkhorst-Pack *k*-point grid with dimensions of 7 × 1 × 2 was employed for Brillouin zone sampling, with a cutoff energy of 500 eV. Convergence was achieved by optimizing the structures until the residual forces on each atom fell below 0.01 eV $Å^{-1}$. Climbing image nudged elastic band (CI-NEB) calculations were conducted to investigate the migration barriers of Mg ions. [S13] The crystal structure is depicted and analyzed using the Visualization for Electronic and Structural Analysis (VESTA) software. [S14]

Supplementary references

S1. X. Deng, Y. Xu, Q. An, F. Xiong, S. Tan, L. Wu, L. Mai, *J. Mater. Chem. A* **2019**, 7, 10644.

S2. D. Bin, W. Huo, Y. Yuan, J. Huang, Y. Liu, Y. Zhang, F. Dong, Y. Wang, Y. Xia, *Chem* **2020**, 6, 968.

S3. W. Yang, L. Dong, W. Yang, C. Xu, G. Shao, G. Wang, Small Methods **2019**, 4, 1900670.

S6. F. Liu, T. Wang, X. Liu, L. Z. Fan, *Adv. Energy Mater.* **2020**, 11, 2000787.

S7. W. Zhou, J. Chen, M. Chen, A. Wang, A. Huang, X. Xu, J. Xu, C.-P. Wong, *J. Mater. Chem. A* **2020**, 8, 8397.

S8. X. Zhang., R. Frech, Electrochim. Acta **1997**, 42, 475.

S9. G. Kresse, J. Furthmüller, *Phys. Rev. B* **1996**, 54, 11169.

S10. P. E. Blöchl, *Phys. Rev. B* **1994**, 50, 17953.

S11. V. I. Anisimov, J. Zaanen, O. K. Andersen, *Phys. Rev. B* **1991**, *44*, 943.

S12. L. Wang, T. Maxisch and G. Ceder, *Phys. Rev. B* **2006**, 73, 1–6.

S13. G. Henkelman, B. P. Uberuaga, H. Jónsson, *The Journal of Chemical Physics* **2000**, *113*, 9901.

S14. K. Momma, F. Izumi, *J Appl Crystallogr* **2011**, *44*, 1272.
